# Surgical Management of Pediatric Head and Neck Sarcoma: A Single-Centre Retrospective Analysis over a 10-Year Period

**DOI:** 10.3390/jcm15093467

**Published:** 2026-05-01

**Authors:** Patryk Kołodziejski, Aleksandra Kołodziejska, Tomasz Brzeski, Maciej Borowiec, Łukasz Krakowczyk, Marcin Kozakiewicz, Krzysztof Dowgierd

**Affiliations:** 1 Department of Maxillofacial and Reconstructive Surgery for Children and Adolescents, Regional Specialized Children’s Hospital, 18a Zolnierska Str., 10-561 Olsztyn, Poland; 2 Department of Conservative Dentistry, Faculty of Medicine, Medical University of Gdańsk, 3c Sklodowska-Curie Str., 80-210 Gdansk, Poland; 3 Department of Pediatric Oncology and Hematology, University of Warmia and Mazury in Olsztyn, Regional Specialized Children’s Hospital, 18a Zolnierska Str., 10-561 Olsztyn, Poland; 4 “Klinika Nieborowice Sp. z o.o.”, 5 Kasztanowa Str., 44-144 Nieborowice, Poland; 5 Department of Maxillofacial Surgery, Medical University of Lodz, 251 Pomorska Str., 92-213 Lodz, Poland; 6 Head and Neck Surgery Clinic for Children and Young Adults, Department of Clinical Pediatrics, University of Warmia and Mazury in Olsztyn, Regional Specialized Children’s Hospital, 18a Zolnierska Str., 10-561 Olsztyn, Poland

**Keywords:** pediatrics, child, sarcoma, surgical oncology, free tissue flaps, therapy, survival

## Abstract

**Objectives:** This study evaluates the epidemiological characteristics and survival, functional, and esthetic outcomes of pediatric patients diagnosed with head and neck sarcoma (PHNS) who underwent individualized surgical treatment for local disease control and/or for defect reconstruction. **Methods:** A cohort of 45 patients aged 0–18 years with histologically confirmed PHNS who underwent surgical resection and/or reconstructive procedures was analyzed. Extracted variables included demographic data, tumor histology and stage, surgical margin status, and systemic therapy modalities. Reconstructive strategies were assessed, considering technique, sequencing, and total duration of treatment. Survival analysis was performed, focusing on both overall survival (OS) and event-free survival (EFS). **Results:** Rhabdomyosarcoma constituted the predominant diagnosis (19/45), followed by Ewing sarcoma (7/45) and chondrosarcoma (5/45). The maxilla represented the most common primary site (18/45), whereas orbital origin was the least frequent (3/45). Complete surgical excision (R0) was achieved in 80.5% of resected cases. Margin status showed no statistically significant association with final outcome (*p* = 0.7786). In contrast, nodal metastasis, local recurrence, and distant dissemination were independently and collectively correlated with mortality. Survival analysis demonstrated a 3-year OS of 100% and an EFS of 79.8%, and a 5-year OS of 94.7% with an EFS of 70.7%. **Conclusions:** Implementation of an individualized surgical and reconstructive protocol was associated with effective local tumor control and favourable reconstructive outcomes. Oncologic prognosis was driven primarily by nodal involvement and recurrent or metastatic disease rather than margin status alone.

## 1. Introduction

Sarcomas are a group of heterogeneous solid tumors derived from mesenchymal tissue [1]. In view of different staging and treatment systems, we distinguish two main groups of sarcomas: soft tissue sarcomas (STSs) and bone sarcomas (BSs) [2]. There are more than 100 different histological types of sarcomas according to the World Health Organization (WHO) [3]. In addition to the type of involved tissue and histological subtype of sarcoma, the affected site is clinically significant.

Because of the anatomical complexity of the afflicted region, pediatric head and neck sarcomas (PHNSs) have different staging, prognosis, and treatment protocols. Due to the complex anatomy, obtaining adequate surgical margins tends to be difficult and may lead to post-therapeutic functional impairment [4,5,6]. I In pediatric patients, sarcomas are the fourth-most frequent head and neck malignancy after lymphoma, neural tumors, and thyroid malignancies, with a prevalence rate of 12% [7].

In non-head and neck sarcomas, metastases are the main cause of mortality. However, in head and neck sarcomas, the main cause of treatment failure is local recurrence [8,9,10,11].

Despite advances in oncologic chemo- and radiotherapy protocols resulting in significant survival improvements, surgery still has a crucial role in local disease control. In selected sarcoma types, surgery remains the main method of treatment.

Surgical treatment methods of head and neck sarcomas have evolved over the years. Initially, patients were treated with resection only, and defects were camouflaged by prosthetic appliances [12]. Later, local flaps were introduced [13,14]. The earliest attempts at microsurgical facial reconstruction in children were published in the 1980s [15,16]. Currently, reconstructions of large defects with microvascular free flaps represents the gold standard in the field of oncologic defect reconstruction. Recently, computer-aided design and manufacturing have led to improvements in surgical resection and reconstruction accuracy. These advances have reduced complications, shortened surgery duration, and improved esthetic and functional outcomes of reconstruction [17,18,19,20,21,22].

The literature in the field of surgical treatment of adults with head and neck sarcoma is substantial. However, reports concerning the pediatric population are still uncommon. A challenge in reporting and analyzing surgical treatment methods is the rarity of the disease and its heterogeneous characteristics. Additionally, especially in the head and neck region, the selection of the local disease control method remains a cause of dispute in the literature. Some authors consider surgical therapy in the head and neck region to be presumably mutilating and functionally debilitating and suggest radiotherapy as an alternative option for selected PHNS cases [23,24]. Others suggest post-radiotherapeutic growth retardation, resultant progressive facial deformation, and more challenging secondary postirradiation reconstruction as indicators for surgery [25,26].

It is vital to define standardized surgical protocols for tumor resection and facial reconstruction in the pediatric population, which has a particularly long-life expectancy and requires highly esthetic and functional reconstruction. Such standards should provide social acceptance for patients who undergo the treatment.

Given these considerations, it is important to analyze treatment protocols, their details, and their effects on survival, function preservation, and cosmetic results to further refine surgical techniques. The rarity of PHNS and the difficulty in reporting sufficiently homogeneous data make every report valuable for future large-scale studies and meta-analyses.

The aim of this study was to retrospectively analyze the epidemiology, survival, and surgical treatment course in a group of pediatric patients managed surgically over a 10-year period, from 2014 to 2024, by a team of maxillofacial surgeons at a reference centre for children.

## 2. Materials and Methods

### 2.1. Data Collection

This study adhered to the STROBE reporting guidelines. Ethical approval was obtained from the Institutional Review Board of the Regional Medical Council of Warmia and Mazury, Poland (L.Dz.WMIL-KB/68/2025; 4 September 2025). The study was conducted in compliance with the principles of the Declaration of Helsinki (59th revision, 21 October 2008). A retrospective cohort design was used to analyze pediatric patients diagnosed with head and neck sarcomas who received treatment at the Regional Specialist Children’s Hospital in Olsztyn (a tertiary referral centre in Poland), between 1 January 2014 and 1 January 2024.

The electronic hospital database was searched for the specified period using the International Statistical Classification of Diseases and Related Health Problems, 10th Revision (ICD-10) diagnostic codes: C00–C14, C41.0, C41.1, C41.8, C41.9, C44.0–C44.4, C44.8, C44.9, C46, C47, C49.0, C49.9, C69, C76.0, C76.7, C76.8 and C80. This search was conducted between 6 and 16 September 2025, and initially identified 121 malignant tumors.

Additionally, a manual review of the institutional pathology unit archives (11–30 September 2025) yielded an additional 15 sarcoma cases. After applying the eligibility criteria, 91 cases were excluded, resulting in a final study sample of 45 patients.

Data were collected by two surgeons with a minimum of five years of clinical experience, under the oversight of pediatric oncology specialists. A senior maxillofacial surgeon independently verified tumor staging and treatment classification to ensure accuracy.

Patients aged 0–18 years with histologically confirmed head and neck soft tissue or bone sarcomas who had completed treatment with curative intent and/or underwent primary or secondary reconstruction at the study centre were included in the analysis. Patients were excluded if their medical records were incomplete, if they received exclusively palliative treatment due to extensive disease or metastases, or if they presented with recurrent tumors initially treated at other centres.

Collected data included patient demographics, tumor histology, anatomical site, clinical stage (according to the American Joint Committee on Cancer [AJCC], 8th edition, and the Children’s Oncology Group [COG]), primary tumor size (mm), age at resection, surgical margin status (R0, R1, R2), and use of chemotherapy and radiotherapy. All histopathologic diagnoses were confirmed by reviewing slides and paraffin blocks. Surgical margins were classified according to the R classification: R0 (no tumor cells in microscopic evaluation), R1 (presence of microscopic tumor infiltration), and R2 (visible macroscopic tumor infiltration at the specimen margin) [27].

Surgical charts were analyzed to determine reconstruction type, sequencing, duration, and microsurgical flap selection. Temporomandibular joint (TMJ) involvement and associated reconstructive strategies were documented. Follow-up data were recorded, including overall survival (OS) and event-free survival (EFS). Serial photographic documentation across all reconstructive stages was examined.

### 2.2. Surgical Treatment Protocol

All patients were treated according to a standardized institutional protocol. Preoperative evaluation of tumor extent included computed tomography (CT), CT angiography, magnetic resonance imaging (MRI), and scintigraphy. For osseous involvement, individualized resection plans were developed, incorporating primary reconstruction of anticipated defects when feasible. Patient-specific cutting guides and osteosynthesis plates were fabricated to ensure precise resections with a planned minimum margin of 0.5 cm, reduce complication rates, and optimize reconstruction outcomes.

For composite bony and soft tissue defects, chimeric flaps were used. Extensive or complex defects occasionally required additional flap modifications, such as segmental osteotomies and the double-bar technique. Additionally, for patients with TMJ involvement, patient-specific TMJ prostheses were fabricated.

For soft tissue sarcomas without osseous extension, comparable presurgical imaging was performed. Resections aimed to achieve at least 0.5 cm of uninvolved tissue, with larger margins when anatomically feasible. Potential donor sites for soft tissue flaps were evaluated to ensure adequate volumetric and esthetic reconstruction of the recipient site and to enable simultaneous work by the resection and reconstruction teams.

This approach aimed to improve recipient site outcomes and reduce operative time through concurrent work by both teams.

### 2.3. Follow Up

Patients who underwent surgical treatment at our unit from 2014 to 2024 were followed-up with pediatric oncologists at the supervising oncology centre according to the Cooperative Weichteilsarkom Studiengruppe (CWS), European and American Osteosarcoma Studies Group (EURAMOS), or European and North American Ewing Sarcoma Study Group (EWING—2008) protocols, depending on tumor type and stage. Parallel follow-up at the study institution included an initial clinical examination at three months postoperatively, followed by evaluations at six-month intervals, with the first postoperative CT or MRI performed at six months. Annual assessments were conducted from three to five years.

### 2.4. Data Analysis

All 45 patients were included in the overall analysis. To evaluate the effectiveness of surgical disease control, a subset of 38 patients who underwent primary tumor resection at the study institution was analyzed separately. Reconstructive outcomes were assessed in the full cohort and further stratified into primary and secondary reconstruction subgroups.

Patient demographics, tumor characteristics, treatment modalities, and outcomes were summarized using descriptive statistics. Continuous data were reported as mean ± standard deviation (SD) or median with interquartile range (IQR), based on the distribution normality determined using the Shapiro–Wilk test. Categorical variables were expressed as absolute numbers or percentages.

Between-group comparisons were conducted using chi-square tests for categorical variables and independent *t*-tests for continuous variables. Correlations were examined through regression analyses. Variables with *p* ≤ 0.1 in bivariate testing were entered into multivariate logistic regression models, with backward elimination applied using a threshold of *p* < 0.157 [28]. The final model was tested for interaction terms, and proportionality assumptions were verified, where a two-tailed *p*-value < 0.05 was considered statistically significant.

Survival outcomes were analyzed using Kaplan–Meier methodology. OS was defined as the time from treatment initiation to death from any cause, and EFS as the interval from treatment initiation to the first documented relapse or death. Five-year survival curves were generated.

## 3. Results

### 3.1. Demographic Findings

Forty-five children with PHNS were identified, including twenty-seven males and eighteen females. Despite the male predominance, no statistically significant correlation between sex and sarcoma incidence was observed. The mean age at diagnosis was 8.35 years (±4.95 years), but the youngest diagnosed patient was 5 months old. Two distinct age ranges associated with higher morbidity were identified: the first between 1 and 5 years of age, and the second between 11 and 16 years of age.

Rhabdomyosarcoma represented the most common malignancy (19/45), followed by Ewing sarcoma (7/45) and chondrosarcoma (5/45). Considering the primary tumor site, the maxilla was the most frequent location (16/45), whereas the orbit was the least common (2/45). Demographic data are summarized in Table 1.

### 3.2. Surgical and Oncologic Tumor Treatment

Of the 45 patients, 38 underwent surgery for local disease control, while 3 had undergone surgery at another centre before referral to the study institution for reconstructive management. The remaining four patients received systemic therapy alone and were referred solely for reconstruction. No cases of familial cancer predisposition syndromes were identified.

In the surgically treated subgroup, 16 of 38 patients presented with stage I disease, 13 with stage II, 3 with stage III, and 6 with stage IV disease. The maximum tumor diameter, as measured on computed tomography imaging, ranged from 12 to 80 mm, with a mean size of 39.9 mm (±14.1 mm).

Postoperative histopathological examination among surgically treated patients demonstrated R0 margins in 29 cases, R1 margins in 8 cases, and R2 margins in 1 case. Of the non-radical resections, three patients presented with stage IV disease, and two were preoperatively misclassified as benign neoplasms based on histopathologic assessment of biopsy material.

Lymphadenectomy of variable extent was performed in 12 patients. Margin status showed no statistically significant association with final outcome (alive/deceased) (*p* = 0.7786).

Chemotherapy was administered to 42 patients, including neoadjuvant therapy in 26, and adjuvant therapy in 9 patients. Seven patients underwent radical chemotherapy combined with radiotherapy as the sole treatment modality, and one patient received palliative chemotherapy.

Radiotherapy was administered to twenty-one patients, with five receiving it as the sole modality of local disease control.

Treatment-related variables are presented in Table 2.

Local recurrence occurred in six patients, and metastatic disease was diagnosed in another six cases. At the time of analysis, forty-two out of forty-five patients remained alive, five were undergoing treatment for tumor recurrence, and three had died.

Bivariate regression analysis of potential predictors influencing the patient’s final outcome identified five variables with *p* ≤ 0.1 (T stage, nodal metastases, distant metastases, secondary oncologic treatment, and local recurrence). Analysis of the variables is presented in Table 3. These predictors were subsequently included in the multivariate analysis.

Additionally, some of these variables showed a statistically significant association with the outcome (*p* < 0.05). Analysis identified significant correlations between outcome (patient survival status: alive/deceased) as the dependent variable and several factors: T status (*p* = 0.0273), nodal involvement (N status) (*p* = 0.0171), receipt of second-line oncologic treatment (*p* = 0.0171), local tumor recurrence (*p* = 0.0003), and late distant metastases (*p* = 0.0415). These predictors were subsequently included in the multivariate analysis.

The final multivariate analysis model revealed a statistically significant association between actual survival status (dependent variable) and the combined effect of nodal involvement, local recurrence, and distant metastases (*p* = 0.0001). The results are presented in Table 4.

**Figure 1:**
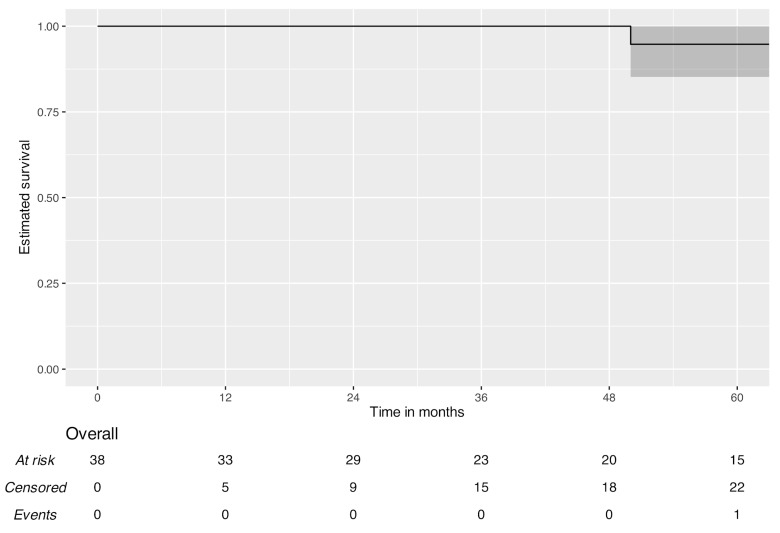
Five-year overall survival.

**Figure 2:**
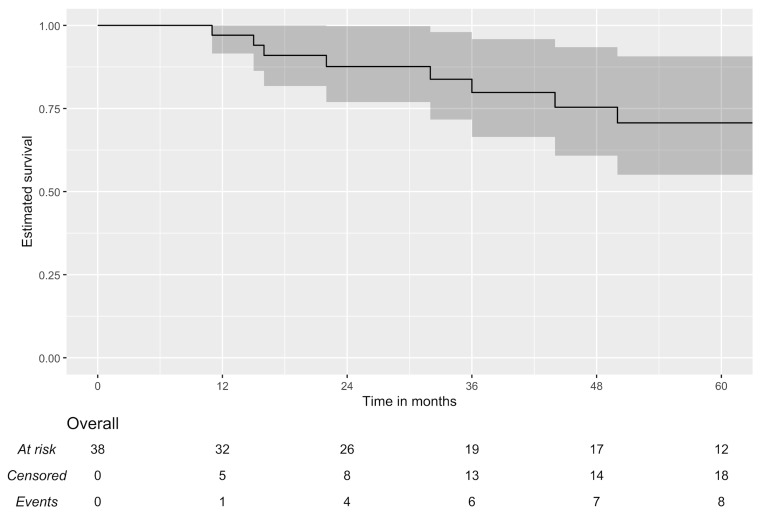
Five-year event-free survival.

**Figure 3:**
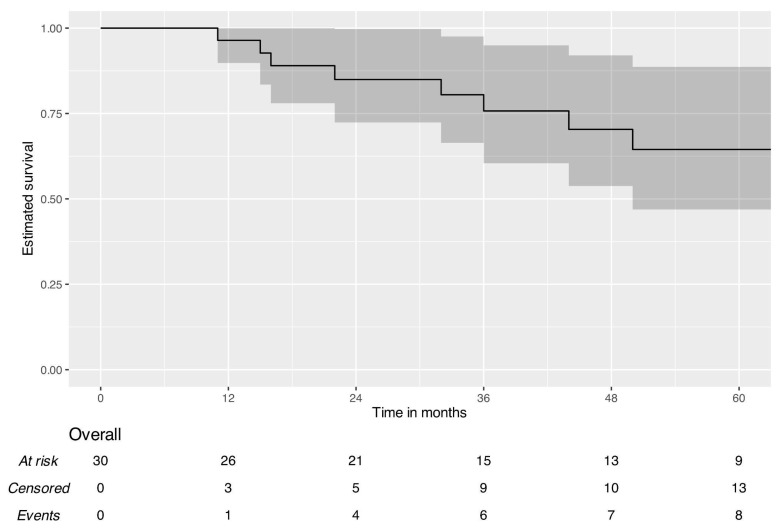
Five-year event-free survival—patients treated with microvascular flap reconstructions.

Survival analysis of patients who underwent surgery for local disease control demonstrated a three-year OS of 100% (95% CI: 100.0–100.0%) and an EFS of 79.8% (95% CI: 66.4–95.9%). The five-year OS was 94.7% (95% CI: 85.2–100.0%), and the EFS was 70.7% (95% CI: 55.1–90.7%). Figure 1, Figure 2 and Figure 3 present five-year Kaplan–Meier plots of OS and EFS, and detailed survival metrics are provided in Table 5.

### 3.3. Reconstructive Treatment

The majority of patients required facial reconstruction. Primary reconstruction was performed in twenty-two cases, while twelve underwent secondary reconstruction, and two required both primary and secondary procedures. Two patients were still waiting for their treatment at the time of this analysis.

Free flap reconstruction was the most frequently employed method (27 cases), followed by local flaps (three cases) and TMJ prosthetic reconstruction combined with free flap reconstruction (three cases). There was one case of isolated TMJ prosthetic reconstruction and one case of alloplastic defect reconstruction using a PEEK (Polyetheretherketone) implant.

Among patients who underwent microvascular reconstruction, three flaps failed due to necrosis, each necessitating reoperation with a new free flap. Donor-site complications were documented in six cases of free flap reconstruction.

At the time of analysis, 18 patients had completed their reconstructive treatment, with a mean therapy duration of 29.9 months (±35.4 months). The remaining 22 patients continued to undergo staged reconstructive procedures.

Detailed reconstructive treatment characteristics are presented in Table 6.

## 4. Discussion

Observed demographic characteristics, histopathological subtypes, and site distribution were comparable to those reported in previously published studies. The age-related morbidity peaks identified in the present analysis are consistent with patterns described in earlier reports [29].

Analysis of postsurgical specimens demonstrated a high rate of R0 resections, with clear margins achieved in 80% of cases after excluding misdiagnosed and underdiagnosed patients. The study by Daw et al. reports R0 resection at the level of 28%. Another research conducted by Tzelnick et al. reports the R0 status around 6%. Additionally, comparing with other similar series available in the literature, this finding suggests improved local tumor control through surgical intervention [23,30,31,32]. This outcome is likely attributable to individualized surgical planning and the integration of microvascular reconstructive techniques, which enhance resection precision. However, the absence of a statistically significant association between margin status and overall outcome indicates that complete resection alone may not be the sole determinant of treatment success. This interpretation should be viewed cautiously, given the heterogeneity of the cohort and the low mortality rate, which may limit statistical power. Additionally, the presence of censored observations may have influenced the survival estimates. As noted previously, the follow-up duration was variable and, in some cases, relatively short. Further research involving larger cohorts or multicenter studies is warranted to more accurately evaluate the impact of surgical clearance on survival within this population. In contrast, large multicentre studies of Dudhat et al. and Moshal et al. have demonstrated the prognostic significance of surgical margins, particularly in sarcomas with limited responsiveness to chemotherapy and radiotherapy [10,33].

The majority of patients received neoadjuvant chemotherapy, which facilitated surgical intervention. Although pediatric cohorts with similar characteristics remain scarce in the recent literature, the observed treatment outcomes are consistent with findings from adult populations and align with contemporary oncologic management strategies presented by Brady et al. and Gradoni et al. [34,35].

Patients who underwent irradiation prior to defect reconstruction, or who received radiotherapy as the sole method of local disease control, experienced increased surgical morbidity and inferior functional and esthetic outcomes compared with non-irradiated patients. These findings are consistent with the study of Park et al. and Radzikowska et al. [36,37] and suggest that preoperative radiation therapy negatively affects reconstructive outcomes.

The results presented in our study are consistent with existing evidence, initial nodal involvement, local recurrence, and metastatic disease were each associated with poorer prognosis, both independently and cumulatively presented in studies of Dudhat et al. and Park et al. [10,36].

In our study, the three-year OS was 100% and the three-year EFS was 79.8%. Moreover, the five-year overall survival was 94.7%, and the three-year event-free survival was 70.7%. Survival outcomes in our study were moderately favourable when compared with data from comprehensive SEER (Surveillance, Epidemiology, and End Results) analyses and single-centre reports. According to Tajudeen et al. a 5-year recurrence-free survival and OS were 50% and 49%. Another study by Peng et al. presents overall two-, five-, and ten-year survival rates for all pediatric sarcoma patients at the level of 84%, 73%, and 71%, respectively. Additionally, Brady et al. report an overall five-year disease-specific survival (DSS) of the investigated group around 80.6% [5,29,32,34,37]. The authors acknowledge limitations related to the small and heterogeneous study cohort. Nevertheless, these outcomes should be verified in larger, multicentre studies.

In the analyzed group, most patients required multistage functional and esthetic reconstruction. As presented above, in the majority of cases, the treatment process extended beyond two years. These findings highlight the need to optimize reconstructive and rehabilitation protocols to improve the efficiency of functional recovery in pediatric patients. While the complexity of pediatric head and neck reconstruction has gained increasing attention in recent years [38,39], reports focusing specifically on reconstructive strategies for PHNS remain limited [40].

An additional challenge concerns patients with TMJ involvement. In this subgroup, joint alloplasty was performed, either alone or in combination with microvascular reconstruction, depending on tumor extent. Despite early postoperative success, long-term outcomes remain uncertain in this group due to subsequent skeletal growth and the extended life expectancy of pediatric patients [41,42,43]. Although the prostheses used were fully patient-specific, their long-term reliability has not been adequately established.

Recipient site morbidity following microvascular reconstruction was comparable to that reported in other clinincal studies by Liu et al. [44]. However, a slightly higher complication rate was observed among the broader pediatric population treated at our institution (including oncologic, trauma, and congenital cases) compared with those reported in adult series. Among potential causes of increased morbidity in the pediatric cohort, neck dissection was considered a factor associated with better outcomes in adults, possibly due to the lower rate of nodal involvement in children. Neck dissection may help prevent vascular pedicle obstruction by providing additional space for tissue edema. The relevance of this procedure in the pediatric population requires further investigation.

Despite the prolonged and complex nature of reconstructive treatment, this study demonstrated satisfactory functional recovery and subjectively acceptable esthetic results. Currently, no validated tools exist for the objective assessment of esthetic outcomes in this population. Given the importance of minimizing patient disfigurement and functional impairment, further assessment of facial function and esthetics is planned and will be presented in future studies with the use of a validated method for the pediatric population.

Regarding the selection of the optimal method for local disease control, the authors presented cases of advanced neoplasms successfully managed with radical surgical excision followed by immediate reconstruction. This approach was associated with better functional and esthetic outcomes and supported a preference for surgical management over irradiation. Pediatric facial irradiation is well known to result in growth retardation, progressive craniofacial deformity, and soft tissue injury [25,26]. Delayed reconstruction following radiotherapy poses substantial clinical challenges in restoring facial symmetry and function.

Based on individualized operative planning, the integration of patient-specific solutions, and the use of advanced microvascular reconstructive techniques, the surgical treatment strategy addressing pediatric patients treated for head and neck sarcomas presents a valuable contribution to the existing literature. In light of the results discussed, the proposed protocol appears to be effective, safe, and advantageous for this patient population.

Finally, the authors acknowledge a high risk of bias in the presented results, primarily due to the small sample size, which reflects the rarity of PHNS and limits the statistical power of the findings. This limitation is further compounded by the retrospective study design, which inherently introduces potential selection bias and reduces control over confounding variables. Additionally, the relatively short follow-up period in some patients may not fully capture long-term outcomes or late complications.

Furthermore, the study cohort is heterogeneous in several important aspects, including patient age, affected anatomical structures, and overall cohort characteristics. The inclusion of patients with different diagnoses makes direct comparisons more challenging. This heterogeneity is also reflected in the use of varying treatment protocols, which may independently influence outcomes and complicate the interpretation of results. Collectively, these factors limit the ability to draw definitive conclusions and highlight the need for larger, prospective, and more standardized studies.

## 5. Conclusions

Pediatric STS and BS are rare and heterogeneous neoplasms, exhibiting distinct histopathological characteristics and tumor-type distributions compared with their counterparts in adults.

The application of microvascular free-tissue transfer and virtual surgical planning during primary tumor resection was correlated with improved oncological and functional outcomes, particularly in achieving clear resection margins, optimizing postoperative functional restoration, and improving patient survival. Statistical analysis of margin status did not demonstrate a significant impact on the patients’ final outcome.

The final outcome of patients (alive or deceased) was influenced by several factors, including nodal metastasis, local recurrence, distant dissemination, and the interplay of these variables.

PHNS frequently results in complex structural and functional deficits, requiring comprehensive reconstructive and rehabilitative strategies. Functional and esthetic restoration is often a prolonged process that may extend over several years to achieve satisfactory results for both patients and clinicians.

Early diagnosis combined with contemporary reconstructive methods and systemic therapy contributes to favourable prognostic outcomes in children diagnosed with PHNS.

## Figures and Tables

**Table 1:** Demographic findings.

	N	%
**Gender**		
M	27	60
F	18	40
Age *		
0	1	2.6
1–5	14	36.8
6–10	8	21.1
11–15	11	29
16–18	4	10.5
**Site**		
Mandible	11	24.4
Maxilla	16	35.6
Orbit	2	4.4
Parameningeal	9	20
Soft tissue	7	15.6
**Histology**		
Rhabdomyosarcoma	19	42.2
Ewing Sarcoma	7	15.6
Chondrosarcoma	5	11.1
Osteosarcoma	5	11.1
Others **	9	20

* Age of patients treated surgically. ** Spindle cell sarcoma, fibromyxoid sarcoma, malignant rhabdoid tumor, alveolar soft part sarcoma, synovial sarcoma, fibrosarcoma.

**Table 2:** Systemic treatment modalities.

	N	%	Status (Alive/Deceased or Progression)
**Surgery Alone**			
Received	3	6.7	
Not received	42	93.3	3/0
**Neoadjuvant + Adjuvant Chemotherapy**			
Received	26	57.8	19/7
Not received	19	42.2	
**Neoadjuvant Radiotherapy**			
Received	0	0	
Not received	45	100	0/0
**Adjuvant Chemotherapy**			
Received	5	11.1	
Not received	40	88.9	5/0
**Combined Adjuvant RT + CT**			
Received	3	6.7	3/0
Not received	42	93.3	
**RT + CT Alone**			
Received	5	11.1	5/0
Not received	40	88.9	
**Chemotherapy Alone**			
Received	2	4.4	1/1
Not received	43	95.6	
**Radiotherapy Alone**			
Received	0	0	
Not received	45	100	0/0

CT—Chemotherapy; RT—Radiotherapy.

**Table 3:** Bivariate logistic regression analysis of potential predictors of outcome of surgical treatment.

Variable	Coefficient *β*	Standard Error	Odds Ratio	*p*-Value
Gender	0.307485	1.2644	1.36	0.8053
Age	−0.0337873	0.122007	0.966777	0.7811
Tumor histology				0.6828
Rhabdomyosarcoma	3.15398 × 10^−9^	387.3	1.0	
RMS-like	3.18057 × 10^−9^	273.863	1.0	
Osteosarcoma	3.26626 × 10^−9^	264.576	1.0	
Ewing sarcoma	−15.2147	223.609	2.46787 × 10^−7^	
Chondrosarcoma	−16.4675	223.611	7.05107 × 10^−8^	
Non RMS-like	3.23589 × 10^−9^	258.2	1.0	
T stage	20.1311	316.23	5.53107 × 10^8^	0.0273 *
Nodal involvement	3.17805	1.36422	24.0	0.0171 *
Metastases	2.61007	1.31507	13.6	0.0415 *
R0 resection	0.367725	1.28435	1.44444	0.7786
Lymphadenectomy	0.0870114	1.27772	1.09091	0.9459
Tumor biggest size (mm)	−0.0573062	0.0493662	0.944305	0.2442
Secondary Margins Excision	−14.2307	158.116	6.60196 × 10^−7^	0.4038
Reconstruction Sequence				0.8072
Primary	0.646627	1.46237	1.90909	
Secondary	13.1682	223.61	523,451	
Reconstruction Type	13.8581	182.577	1.04346 × 10^6^	0.7414
Chemotherapy administration	13.0271	182.576	454,576	0.5074
Chemotherapy Type				0.7449
Neoadjuvant	−14.0812	223.609	7.6668 × 10^−7^	
Adjuvant	−14.4867	223.61	5.1112 × 10^−7^	
Radical	−1.06087 × 10^−9^	264.576	1.0	
Radiotherapy	−0.492476	1.26525	0.611111	0.6912
Secondary oncologic treatment	3.17805	1.36422	24.0	0.0171 *
Local Recurrence	19.5661	52.7112	3.14386 × 10^8^	0.0003 *

* *p* < 0.05.

**Table 4:** Multivariable logistic regression analysis of fatality risk factors in sarcoma treatment in the pediatric population.

Variable	Coefficient *β*	Standard Error	χ^2^ Value	Odds Ratio	*p*-Value
N	24.48	159	4.499	3.83 × 10^10^	0.0339 *
Local Recurrence	48.83	145	13.392	1.62 × 10^21^	0.0003 *
M1	23.73	135	4.499	2.02 × 10^10^	0.0339 *

N—nodal involvement; M1—distant metastasis; * *p* < 0.05.

**Table 5:** One-, three-, five-, and ten-year event-free survival.

Time	At Risk	Events	Survival	95% Confidence Interval	
				Lower	Upper
12	33	1	97.1%	91.5%	100.0%
36	21	5	79.8%	66.4%	95.9%
60	12	2	70.7%	55.1%	90.7%
120	4	1	58.9%	38.1%	91.1%

**Table 6:** Reconstructive treatment modalities.

	N	%	Completion of Reconstructive Treatment (Yes/No)
**Local Flap**			
Received	2	5.6	1/1
Not received	34	94.4	
**Regional Flap**			
Received	0	0	0/0
Not received	36	100	
**Microvascular Reconstruction**			
Received	28	77.8	13/15
Not received	8	22.2	
**TMJ Prosthesis Alone**			
Received	1	2.8	0/1
Not received	35	97.2	
**MR + TMJ Prosthesis**			
Received	4	11.1	2/2
Not received	32	88.9	
**PEEK Implant**			
Received	1	2.8	1/0
Not received	35	97.2	

TMJ Prosthesis—Temporomandibular joint prosthesis; MR—Microvascular reconstruction; PEEK—Polyetheretherketone.

## Data Availability

Publicly available data are provided within the manuscript. Data which are unavailable due to privacy or ethical restrictions can be requested from P.K.

## Abbreviations

The following abbreviations are used in this manuscript:

STSs	Soft Tissue Sarcomas
BSs	Bone Sarcomas
WHO	World Health Organization
PHNSs	Pediatric Head and Neck Sarcomas
ICD-10	International Statistical Classification of Diseases and Related Health Problems, 10th Revision
AJCC	American Joint Committee on Cancer
COG	Children’s Oncology Group
TMJ	Temporomandibular joint
OS	Overall Survival
EFS	Event-free Survival
CT	Computed Tomography
MRI	Magnetic Resonance Imaging
CWS	Cooperative Weichteilsarkom Studiengruppe
EURAMOS	European and American Osteosarcoma Studies Group
EWING-2008	European and North American Ewing Sarcoma Study Group
SD	Standard Deviation
IQR	Interquartile Range
PEEK	Polyetheretherketone
SEER	Surveillance, Epidemiology, and End Results
